# Respiratory distress in small for gestational age infants based on local newborn curve prior to hospital discharge

**DOI:** 10.3389/fped.2022.986695

**Published:** 2022-09-30

**Authors:** Ekawaty Lutfia Haksari, Mohammad Hakimi, Djauhar Ismail

**Affiliations:** ^1^ Department of Child Health, Faculty of Medicine Public Health and Nursing, Universitas Gadjah Mada, Sardjito General Hospital, Yogyakarta, Indonesia; ^2^ Faculty of Medicine Public Health and Nursing, Universitas Gadjah Mada, Yogyakarta, Indonesia

**Keywords:** respiratory distress, prior to hospital discharge, preterm-SGA, local newborn curve, length of stay, ventilator use

## Abstract

**Background:**

Respiratory distress in newborns, which may lead to risks of morbidity and death, is one of the reasons for a referral to the more advanced health facilities. Respiratory distress analysis in small for gestational age (SGA) infants remains controversial. SGA infants are a big problem for and burden the low-medium income countries. Frequentness of SGA infants varies, depending on birth weight curve used.

**Objective:**

To identify the risks, complications, death induced by respiratory distress in SGA infants prior to hospital discharge.

**Methods:**

A retrospective cohort study was conducted on live- born infants at Sardjito Hospital. Singleton and gestational age 26–42 weeks were the inclusion criteria. The exclusion criteria included major congenital anomaly, chromosomal abnormalities, out-born infants admitted >24 h, discharge against medical advice, and incomplete data. The samples were categorized into appropriate gestational age (AGA) and SGA by a local newborn curve. The samples were also classified as full-term (FT)-AGA, preterm (PT)-AGA, FT-SGA, and PT-SGA. Odds ratio (OR) was based on entire respiratory distress. Complications of respiratory distress analyzed were length of hospital stay, administration of oxygen, Continuous Positive Airway Pressure (CPAP), and ventilator. Reverse Kaplan-Meier and Cumulative Mortality Incidence (CMI) were used to analyze respiratory distress-induced mortality. Stata 13 was used to analyze the data.

**Results:**

There were 12,490 infants eligible for the study, consisting of 9,396 FT-AGA infants, 2,003 PT-AGA infants, 771 FT-SGA infants, and 320 PT-SGA infants. Nine hundred and thirty-two infants developed respiratory distress. Multiple logistic regression analysis revealed highest risk of respiratory distress in PT-SGA infants with OR 5.84 (4.28–7.99). The highest respiratory distress complications were found in PT- SGA with significant difference on length of hospital stay and IRR 2.62 (2.09–3.27). In addition, the highest use of mechanical ventilator was found in PT-SGA with significant difference. CPAP use was the highest in PT-AGA infants. There was no significant difference in oxygen administration among the groups. Respiratory distress-induced mortality analysis found the highest CMI in PT-SGA infants.

**Conclusion:**

PT-SGA had the highest risk of respiratory distress with complications of length of hospital stay, and ventilator use. Mortality analysis discovered the highest CMI in PT-SGA infants. We should therefore be alert when dealing with PT-SGA infants.

## Introduction

Respiratory distress in neonates is a threatening condition and the most common reason for hospital reference. This problem requires an immediate assessment, frequent monitoring, and urgent treatment. The management of respiratory distress may succeed if maternal and neonatal history, clinical examination, understanding of associated respiratory distress cases, differential diagnosis, and identification of life-threatening conditions are obtained (1). Early recognition and immediate intervention for this problem will decrease the risk of short-term and long-term complications and reduce the mortality in the neonates at risk (2).

Respiratory distress analysis in small for gestational age (SGA) infants remains controversial. SGA infants are generally considered showing better conditions than those of appropriate gestational age (AGA) infants with the same body weight, due to increased maturity of the lungs. This is crucial for the recognition of complications that may occur in both SGA and AGA infants. With gestational age, the same sex and race, however, SGA infants are actually no better than AGA infants in every analysis. Several analyses reported that SGA infants posed a significant increased risk of respiratory distress syndrome (RDS) and, by most analyses, respiratory problems and death (3). Additionally, SGA infants suffering respiratory distress during the neonatal period are likely to cause mortality and morbidity to the survivor.

An epidemiological review demonstrated increased morbidity and respiratory distress in SGA infants from their neonatal state to their infancy and childhood (4, 5). In addition to becoming a big burden for low-middle income countries, SGA infants are a global problem which is critical to handle, especially in some South Asian countries, some African countries, and other developing countries. Understanding of the problem and performing an effective intervention to very small infants born too early are the chief priorities to improve safety and reduce morbidity, stunting, and non-contagious diseases (6–8). Appropriate preventive actions and management conducted in Tanzania have been proven to decrease infant mortality (9).

Prevalence of SGA infants varies, depending on birth weight curve used (7). Local neonatal curves for birthweight, supine length, and head circumference have been developed in Yogyakarta, Indonesia, to classify high risk newborns and identify newborns requiring attention (10).

## Objectives

To identify the risks, complications, death induced by respiratory distress in SGA infants prior to hospital discharge.

## Materials and methods

### Study population

A retrospective cohort study was conducted on the live-birth infants born and cared at Sardjito General hospital from 1 January 2008 to 31 March 2017. The inclusion criteria were singletons with gestational ages 26–42 weeks, whereas major congenital anomalies, chromosomal abnormalities and out-born infants admitted >24 h, discharge against medical advice, and incomplete data constituted the exclusion criteria.

### Data collection

The dependent variable was respiratory distress during hospital stay, while the independent variable was SGA infants. The data were collected from Sardjito hospital using form of South East Asian Regional Neonatal Perinatal death of World Health Organization and medical records.

Mother's age referred to the age of mother when she gave birth. Mother are at risk of infection if they have premature rupture of membrane >18 h, or fever during peripartum or leukocytosis or urinary tract infection. The delivery methods included spontaneous delivery, assisted to spontaneous delivery, and caesarean section. Maternal death is a condition when a mother dies as being discharged from the hospital. The respiratory distress was clinically assessed with Downes score (11–13) and diagnostic support. Gestational age was estimated in weeks by using Dubowitz score or, if not possible, by Ballard score (14, 15). Sex referred to boy and girl physically. Asphyxia was defined as no breathing at birth. Apgar score in 5 min was <6, or 2 cycles positive pressure ventilation was needed, or PH <7.2 of blood gas analysis was necessary. Sepsis was confirmed clinically when there was at least 1 case in 4 of 6 groups (general condition, cardiovascular, gastrointestinal, respiration, central nervous system, hematology) or there was a positive bacterial culture in the body fluid, by clinical practice guidelines of Sardjito hospital. Meanwhile, resuscitation referred to a condition when the infant required it at birth.

### Statistical analysis

Based on the local newborn curve (10), the infants with more than or equal to 10 percentiles were categorized into AGA and those with less than 10 percentiles were classified as SGA. There were 4 groups of infants by full-term-preterm and AGA-SGA were Full-term (FT)-AGA, Preterm (PT)-AGA, FT-SGA, and PT-SGA. The other risk factors that served as the confounding variables included sex, sepsis, asphyxia, resuscitation, mother's age, risk of mother's infection, delivery method, and death. The frequency distribution was calculated to describe 4 groups of infants and other risk factors.

Odds-ratio (OR) was obtained from the overall respiratory distress in the four groups of infants and the other risk factors using simple logistic regression. Multiple logistic regression analysis was conducted if the result of other risk factors from the simple logistic regression were *p* < 0.25 (16). It was also conducted to identify the confounding variables.

The complications of respiratory distress analyzed during hospital care were length of hospital stay, oxygen administration, use of Continuous Positive Airway Pressure (CPAP) and ventilator, and death. The difference of length of hospital stay among the four groups of infants with FT-AGA as a referral was calculated by negative binomial regression analysis (17). We also noted the proportion of oxygen administration, application of CPAP and ventilator for respiratory distress during hospital stay. Reverse Kaplan Meier method was done to calculate the Cumulative Mortality Incidence (CMI) of the neonates with respiratory distress in the four groups of infants during hospital stay, for which FT- AGA was used as a referral. The value *p* < 0.05 was considered statistically significant. STATA 13 was used to analyze the data.

This study has been approved by Medical and Health Research Ethics Committee Faculty of Medicine Universitas Gadjah Mada–Dr. Sardjito General Hospital (KE/FK/0979/EC/2017).

## Results

### Baseline information

**Figure 1:**
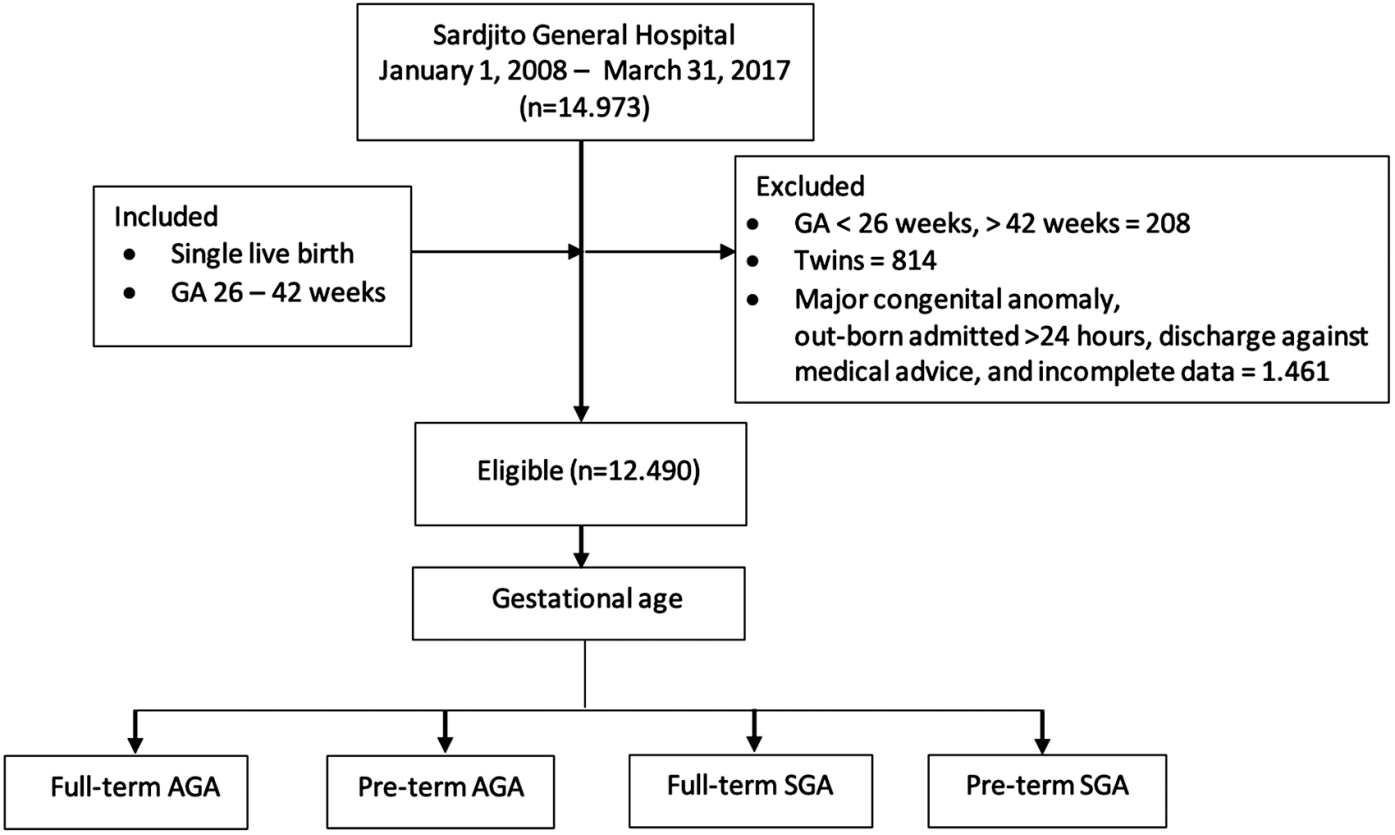
Flowchart of the study.

Of all the neonates who fulfilled the inclusion and exclusion criteria, there were 14,973 with gestational age <26 weeks and 208 with gestational age >42 weeks. Also, there were 814 twins. Meanwhile, 1,461 neonates had congenital anomalies or incomplete data. We extracted 12,490 neonates eligible for the study (Figure 1).

Of all the eligible samples, there were 9,492 (76.0%) infants with normal birth weight and 2,990 infants with low birth weight (LBW) (24.0%). SGA infants constituted 8,7% and AGA infants made up 15,2%. Meanwhile, there were 1,091 (36.5%) SGA infants among those with LBW and 1,899 (63.5%) AGA. There were 2,323 (18.6%) preterm infants and 10,167 (81.4%) full-term infants. We found 9,396 (75.2%) FT-SGA infants by gestational age criteria, 2,003 (16.04%) PT-AGA infants, 771 (6.2%) FT- SGA infants, and 2.56% PT-SGA infants. There was a statistically significant difference distribution of gestational age, sex, asphyxia, resuscitation, mother's age, mother's education, parity, antenatal care (ANC), delivery method, and birth weight among PT-SGA, FT-SGA, PT-SGA and FT-SGA distribution of gestational age, sex, asphyxia, resuscitation, mother's age, mother's education, parity, antenatal care (ANC), delivery method, and birth weight among PT-SGA, FT-SGA, PT-SGA and FT-SGA (Table 1).

**Table 1:** Characteristics of newborn and mother by full-term preterm and AGA-SGA.

Variable	Total	FT-AGA	FT-SGA	PT-AGA	PT-SGA	*p*-value
	*n* = 12,490	*n* = 9,396	*n* = 771	*n* = 2,003	*n* = 320	
Sex						0.001
Boy	6,475 (51.8%)	4,905 (52.2%)	360 (46.7%)	1,066 (53.2%)	144 (45.0%)	
Girl	6,015 (48.2%)	4,491 (47.8%)	411 (53.3%)	937 (46.8%)	176 (55.0%)	
Sepsis						<0.001
Yes	1,705 (13.7%)	576 (6.1%)	167 (21.7%)	776 (38.7%)	186 (58.1%)	
Asphyxia						<0.001
Yes	332 (2.7%)	107 (1.1%)	30 (3.9%)	148 (7.4%)	47 (14.7%)	
Resuscitation						<0.001
Yes	2,279 (18.2%)	1,043 (11.1%)	216 (28.0%)	832 (41.5%)	188 (58.8%)	
Mother's age						<0.001
<18 years	319 (2.6%)	251 (2.7%)	14 (1.8%)	48 (2.4%)	6 (1.9%)	
18–35	10,214 (81.8%)	7,833 (83.4%)	621 (80.5%)	1,509 (75.3%)	251 (78.4%)	
>35	1,957 (15.7%)	1,312 (14.0%)	136 (17.6%)	446 (22.3%)	63 (19.7%)	
Mother's infection risk						<0.001
Yes	437 (3.5%)	216 (2.3%)	25 (3.2%)	175 (8.7%)	21 (6.6%)	
Delivery mode						<0.001
Spontaneous	6,752 (54.1%)	5,291 (56.3%)	376 (48.8%)	987 (49.3%)	98 (30.6%)	
Assisted	596 (4.8%)	521 (5.5%)	22 (2.9%)	47 (2.3%)	6 (1.9%)	
Caesarean	5,142 (41.2%)	3,584 (38.1%)	373 (48.4%)	969 (48.4%)	216 (67.5%)	
Mother's education						<0.001
≦6 years	1,087 (8.7%)	785 (8.4%)	75 (9.7%)	201 (10.0%)	26 (8.1%)	
7–12	1,700 (13.6%)	1,197 (12.7%)	119 (15.4%)	339 (16.9%)	45 (14.1%)	
13+	9,703 (77.7%)	7,414 (78.9%)	577 (74.8%)	1,463 (73.0%)	249 (77.8%)	
Parity						<0.001
≦1	10,119 (81.0%)	7,674 (81.7%)	637 (82.6%)	1,549 (77.3%)	259 (80.9%)	
2–4	2,293 (18.4%)	1,675 (17.8%)	131 (17.0%)	431 (21.5%)	56 (17.5%)	
5+	78 (0.6%)	47 (0.5%)	3 (0.4%)	23 (1.1%)	5 (1.6%)	
ANC						<0.001
0	8 (0.1%)	7 (0.1%)	1 (0.1%)	0 (0.0%)	0 (0.0%)	
1–4	202 (1.6%)	118 (1.3%)	17 (2.2%)	58 (2.9%)	9 (2.8%)	
5+	12,280 (98.3%)	9,271 (98.7%)	753 (97.7%)	1,945 (97.1%)	311 (97.2%)	

AGA, appropriate gestational age, ANC, antenatal care, SGA, small for gestational age, FT-AGA, full-term AGA, FT-SGA, full-term-SGA, PT-AGA, preterm-AGA, PT-SGA, preterm-SGA.

### Identification risks of respiratory distress by simple and multiple logistic regression analysis

Simple logistic regression analysis revealed the highest risk of respiratory distress of infants in PT-SGA with OR 26.6 (20.7–34.2), resuscitation with OR 24.9 (21.1–29.4), sepsis with OR 19.5 (16.8–22.7), asphyxia with OR 17.5 (13.9–22.0), PT-AGA with OR 9.96 (8.49–11.7), risk of mother's infection with OR 4.66 (3.71–5.84), FT-SGA with OR 3,11 (2,34–4,12), caesarean delivery with OR 1.86 (1.62–2.14), mother's age with OR1.52 (1.29–1.79), assisted spontaneous with OR 1.45 (1.06–2.14), and boy with OR 1.16 (1.01–1.33) respectively (Table 2).

**Table 2:** Simple and multiple logistic regression analysis on overall risks of respiratory distress.

		Respiratory distress			
		Simple logistic regression		Multiple logistic regression	
	*n* = 12,490	OR	[CI 95%]	OR	[CI 95%]
Full-term-Preterm, AGA-SGA					
FT-AGA	9,396	1		1	
FT-SGA	771	3.11***	[2.35–4.12]	1,33	[0.96–1.84]
PT-AGA	2,003	9.96***	[8.49–11.7]	2.89***	[2.37–3.51]
PT-SGA	320	26.6***	[20.7–34.2]	5.84***	[4.28–7.99]
Sex					
Boy	6,475	1.16*	[1.01–1.33]	1.17	[0.99–1.39]
Girl	6,015	1		1	
Sepsis					
Yes	1,705	19.5***	[16.8–22.7]	5.56***	[4.66–6.63]
No	10,785	1		1	
Asphyxia					
Yes	332	17.5***	[13.9–22.0]	2.32***	[1.76–3.05]
No	12,158	1		1	
Resuscitation					
Yes	2,279	24.9***	[21.1–29.4]	9.08***	[7.50–11.0]
No	10,211	1		1	
Maternal age					
<18 years	319	0,84	[0.53–1.35]	0,96	[0.54–1.69]
18–35	10,214	1		1	
>35	1,957	1.52***	[1.29–1.79]	1,16	[0.94–1.43]
Maternal infection risk					
Yes	437	4.66***	[3.71–5.84]	2.45***	[1.81–3.31]
No	12,053	1		1	
Delivery mode					
Spontaneous	6,752	1		1	
Assisted					
Spontaneous	596	1.45*	[1.06–1.99]	1.14	[0.77–1.68]
Caesarean	5,142	1.86***	[1.62–2.14]	1.02	[0.86–1.22]

AGA, appropriate gestational age, CI, confident interval, FT, full-term, PT, preterm, SGA, small for gestational age, OR, Odds ratio.

* *p* < 0.05.

** *p* < 0.01.

*** *p* < 0.001.

Multiple logistic regression analysis demonstrated that there were a significant difference and a highest risk in resuscitation with OR 9.08 (7.05–11.0), PT-SGA with OR 5.84 (4.28–7.99), sepsis with OR 5.56 (4.66–6.63), PT-AGA with OR 2.89 (2.37–3.51), mother's risk of infection with OR 2.45(1.81–3.31), asphyxia with OR 2.32 (1.76–3.05) consecutively. In the group of full-term-preterm and SGA-AGA, we found the highest remained in PT-SGA infants with OR 5.84 (4.28–7.99) and in PT-AGA infants with OR 2.89 (2.37–3.51) respectively. Meanwhile OR 1.33 (0.96–1.84) was found in FT-SGA infants, which was insignificantly different from FT-AGA infants (Table 2).

### Complications of respiratory distress analysis during hospital care

Length of hospital stay of the neonates with respiratory distress, by gestational age and AGA-SGA, with FT-AGA as a referral exhibited a statistically significant difference, for which the highest Incidence rate ratio (IRR) was found in PT-SGA by 2.62 (2.09–3.27), followed PT-AGA by 2.17 (1.83–2.56), FT-SGA by 1.49 (1.10–2.04) successively (Table 3).

**Table 3:** Effect of small for gestational age on length of hospital stay among respiratory distress.

Gestational Age	Length of stay					
	*n*	median	IQR	IRR^a^	95% CI	
FT-AGA (reference)	225	6	6	1		
FT-SGA	54	9,5	11	1.49**	1.10	2.04
PT-AGA	426	14	23	2.17***	1.83	2.56
PT-SGA	132	18	28	2.62***	2.09	3.27
Total	837	10	19			

AGA, appropriate gestational age, CI, confident interval, FT, full-term, PT, preterm, SGA, small for gestational age, IQR, Inter Quartile Range, IRR, Incidence Rate Ratio.

^a^ Calculated by Negative Binomial Regression.

** *p* < 0.01.

*** *p* < 0.001.

**Figure 2:**
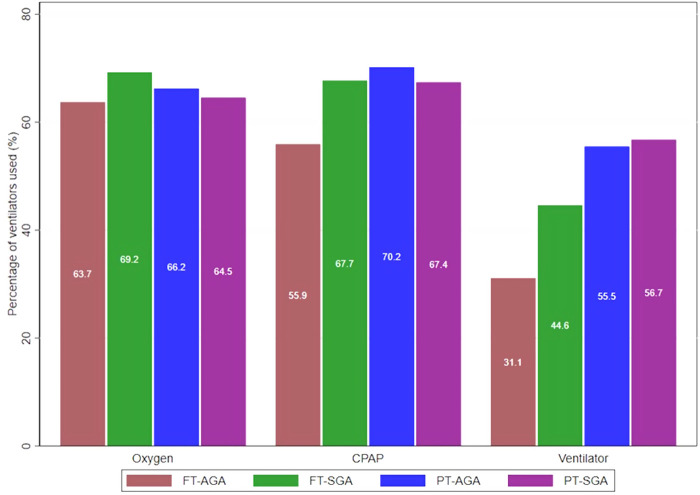
Administration of oxygen, CPAP, ventilator in four groups of full-term-preterm and AGA SGA.

Despite the various types of ventilators used in the study, we decided to make a group of ventilators. The overall respiratory distress was presented in the bar charts. There was a significant difference of overall respiratory distress in CPAP group and ventilator group, with statistically difference. The highest percentage of CPAP use, by gestational age, was identified in PT-AGA infants, whereas the highest percentage of ventilator use was found in PT-SGA. No significant difference was found in oxygen administration (Figure 2).

### Mortality analysis

**Figure 3:**
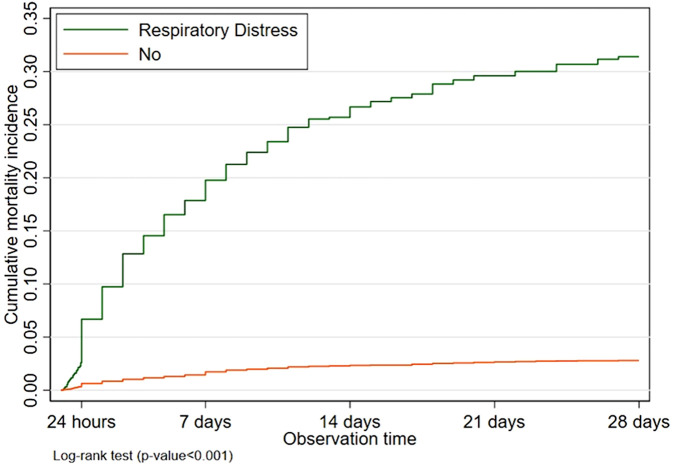
Cumulative mortality incidence of newborns with respiratory distress compared with absence of respiratory distress in neonatal period.

**Figure 4:**
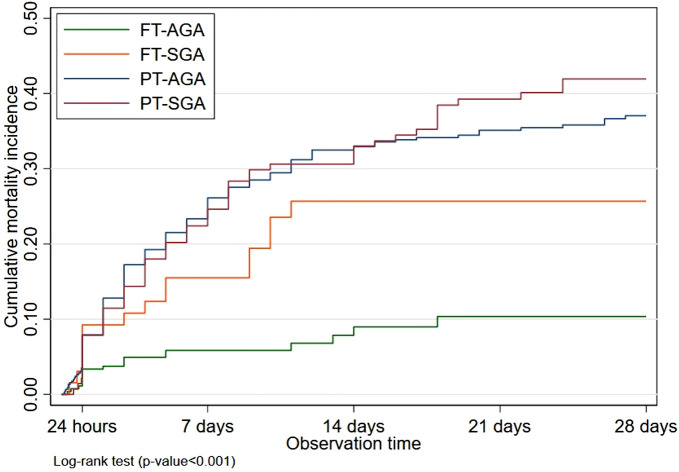
Cumulative mortality incidence of respiratory distress in newborns by full term-preterm and AGA-SGA during neonatal period.

Mortality analysis on the overall respiratory distress showed higher CMI in infants with respiratory distress than in those who did not develop it during the neonatal period, starting from birth until the end of the neonatal period (Figure 3). Based on the gestational age and AGA – SGA, the highest CMI was found in PT-SGA infants, followed by PT-AGA, FT-SGA, and FT-AGA sequentially. The CMI in PT-SGA infants was almost as high as that in PT-AGA infants. At the end of 21 days of age, however, the risk of death increased in PT-SGA infants (Figure 4).

## Discussion

Overall respiratory distress in this study was assessed clinically using Downes score and diagnostic support. Meanwhile, other studies generally discussed the various causes of respiratory distress.

### Risks of respiratory distress

Our study reported that resuscitation, sepsis, asphyxia, and mother's risk of infection were the risk factors for respiratory distress. It was consistent with the recommendation of post – resuscitation monitoring, stating that respiratory distress may occur following neonatal resuscitation and birth asphyxia (18). Sepsis-induced respiratory distress is associated with high mortality in critically ill patients, although the incidence is relatively low. The evaluation of risk factors of developing respiratory distress in patients with severe sepsis is of the utmost importance. Sepsis and respiratory distress share similar mechanisms, although differentiating the indirect injury from the direct injury using a panel of biomarkers may be useful in understanding of sepsis-induced respiratory distress and for selecting patients in trials of new therapies (19). Low birth weight and low serum albumin levels are associated with an increased development of respiratory distress in early onset of sepsis in infants. Early onset of sepsis with respiratory distress is associated with a higher overall disease severity, more severe complications, and higher case-fatality rate (20). Meanwhile, mother's risk of infection is associated with respiratory distress in neonates (1).

In this study, the risk of all respiratory distress based on the full-term-preterm and AGA-SGA, using FT-AGA as reference; was detected the highest in PT-SGA infants, then PT-AGA infants, FT-SGA respectively. Other studies reported various results and conclusions. The results of our study were different from those of the previous studies, which reported lower risk of hyaline membrane disease or also known as respiratory distress syndrome (RDS) in SGA infants. The study, however, compared the groups of AGA-SGA only without using gestational age (21). Other studies claimed that both SGA and AGA infants were at the same risk to develop RDS (22, 23). In the study by Bardin, the infants were classified by AGA and SGA with gestational age <27 weeks and SGA criteria if <3 percentile of birth weight. Sharma, who used gestational age as a control, reported no difference of risks of RDS in SGA and AGA infants with gestational age ≤32 weeks. However, the risk of RDS in SGA infants would significantly decrease at gestational age >32 weeks (24).

Turizt confirmed that SGA was not associated with RDS or other adverse respiratory and neonatal composite. RDS and the composite respiratory outcome were mostly associated with earlier gestational age at delivery, caesarean delivery, and male gender. In her study, the subjects were preterm infants and singletons with gestational ages 22–36 weeks. SGA was defined as below 10 percentiles and their weight was based on Brenner's curve. The composite respiratory outcomes in her study included RDS, transient tachypnea of the newborn, bronchopulmonary dysplasia, and a ventilator-dependent condition, which served as a secondary outcome as well. Meanwhile, the composite adverse neonatal outcome consisted death, sepsis, necrotizing enterocolitis, seizure, and intraventricular haemorrhage (23).

Some studies reported the same result as that of our study, showing that SGA infants were at increased risk of respiratory distress. Tyson also reported that SGA infants increased the risk of RDS and other types of respiratory distress. His study compared SGA and AGA infants by gestational age, sex, and the same race. The criteria of SGA infants in his study were those with birth weight <10 percentiles and gestational age by Canadian Arbucle and Parkland curves (3). Piper et al., 1996 reported that RDS in SGA infants was higher than that in AGA infants. Her study used lower birth weight (25). Zaw stated that PT-SGA infants had an increased risk of RDS and respiratory morbidity. The criteria for SGA infants included birth weight <10 percentiles using Arbucle's neonatal birth weight curve and standard fetal curve of Hadlock (4).

### Complications of respiratory distress

The complications of respiratory distress we studied were length of hospital stay, oxygenation, CPAP, and ventilator. There was a statistically significant difference in length of hospital stay among the neonates who suffered respiratory distress, for which the highest was found in PT-SGA. The results were generally similar to those of the other studies which reported that SGA infants were likely to have a longer hospital stay than AGA infants (22, 24, 26–30). Also, Full-term-SGA infants had a potentially longer hospital stay than AGA infants (31). However, Charles, who compared SGA and AGA on extremely low birth weight, explained similar length of stay of SGA to AGA infants (32).

In our study, based on full-term-preterm and AGA-SGA, all respiratory distress in the four groups displayed no significant difference in the use of oxygen therapy. It was fairly similar to the results of the other studies, reporting that oxygen therapy in SGA infants was practically similar to that in AGA infants (24, 33). The cohort of late preterm birth presented no significant difference in oxygen administration between SGA and AGA (32). However, others studies showed more prolonged need for oxygen supplementation in SGA infants vs. AGA infants born before <29 weeks (22, 26).

This study reported a statistically significant difference in the use of CPAP, in which the highest rate was found in PT-AGA. Meanwhile, other studies reported no significant difference in CPAP use between SGA and AGA infants whose gestational ages were <24 weeks (31), and late preterm (34).

Our study, discovered a statistically significant difference in the use of ventilator. The highest rate of ventilator use was found in PT- SGA infants. Bardin explained more prolonged need of ventilatory support for SGA infants than for AGA infants born before <27 weeks (22). Tannirwar who studied <24 weeks of gestational age infants, however, reported no significant difference in the use of ventilator in PT-SGA. Similar result was also recorded by Barthal, who investigated late preterm infants (33, 34). There was no significant difference between SGA and AGA infants in the total days on ventilator (24).

### Mortality analysis

Our analysis on mortality and by gestational age demonstrated the highest CMI in PT-SGA, PT- AGA, FT-SGA, and FT- AGA respectively. The CMI in PT-SGA was nearly as high as that in PT-AGA. Nevertheless, at the end of 22 days of age PT-SGA infants would increase the risk of mortality. The result of another study presented a similar report in which PT-SGA infants had a higher risk of death (OR 3.1 *p* = 0.001) than AGA infants. The study, however, analyzed the RDS group only (24).

### Weakness and strength of the study

We used the retrospective-cohort which had a potential data loss. The data were collected soon after the infants were born, a standard data collection at hospitals. They were subsequently matched and re-checked for possible incorrectness or incompleteness. Later, a trained staff would enter the data into the database. Additionally, our study only used 1 tertiary hospital despite the high number of samples.

On the other hand, our study was based on a local newborn curve with high number of samples so that we could make an analysis on morbidity and respiratory distress. Moreover, we could compare the risks of SGA infants and their potential complications during hospital stay. We strongly underline the importance of studying SGA infants and the consequences of their clinical death and respiratory distress.

## Conclusion

Multiple regression analysis on the overall respiratory distress, based on full-term-preterm and AGA-SGA by local newborn curve, suggested the high risk of respiratory distress in resuscitated newborns, PT-SGA, septic neonates, PT-AGA, mother's infection risk, and asphyxiated neonates consecutively.

Overall complications of respiratory distress with length of hospital stay were the highest IRR in PT-SGA. In addition, the highest use of mechanical ventilator was found in PT-SGA infants and an analysis on neonatal mortality with the highest CMI in PT-SGA infants. We should therefore be alert when dealing with PT-SGA infants.

## Data Availability

The raw data supporting the conclusions of this article will be made available by the authors, without undue reservation.
